# Multiconfigurational Quantum Chemistry Determinations of Absorption Cross Sections (σ) in the Gas Phase and Molar Extinction Coefficients (ε) in Aqueous Solution and Air–Water Interface

**DOI:** 10.1021/acs.jctc.0c01083

**Published:** 2021-05-11

**Authors:** Ana Borrego-Sánchez, Madjid Zemmouche, Javier Carmona-García, Antonio Francés-Monerris, Pep Mulet, Isabelle Navizet, Daniel Roca-Sanjuán

**Affiliations:** † Instituto Andaluz de Ciencias de la Tierra, CSIC-University of Granada, Av. de las Palmeras 4, 18100 Armilla, Granada, Spain; ‡ MSME, Univ Gustave Eiffel, CNRS UMR 8208, Univ Paris-Est Créteil 8208, F-77454 Marne-la-Vallée, France; § Instituto de Ciencia Molecular, Universitat de València, P.O. Box 22085, València, Spain; ∥ Université de Lorraine and CNRS, LPCT UMR 7019, F-54000 Nancy, France; ⊥ Departamento de Química Física, Universitat de València, C/Dr. Moliner 50, 46100 Burjassot, Spain; # Departamento de Matemáticas Área de Matemática Aplicada Facultad de Matemáticas C/Dr. Moliner, 50 46100 Burjassot, Spain

## Abstract

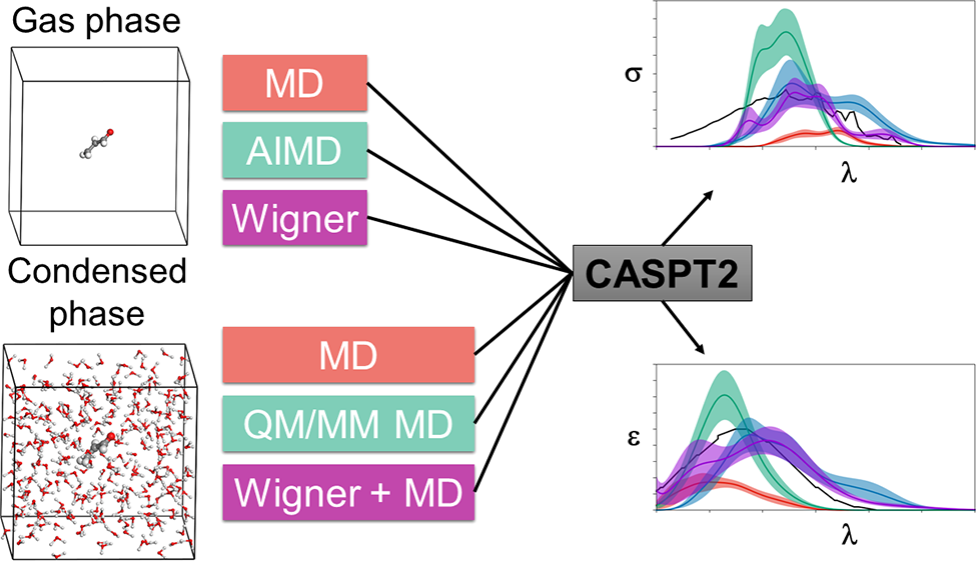

Theoretical determinations of absorption cross sections (σ) in the gas phase and molar extinction coefficients (ε) in condensed phases (water solution, interfaces or surfaces, protein or nucleic acids embeddings, etc.) are of interest when rates of photochemical processes, *J* = ∫ ϕ(λ) σ(λ) *I*(λ) dλ, are needed, where ϕ(λ) and *I*(λ) are the quantum yield of the process and the irradiance of the light source, respectively, as functions of the wavelength λ. Efficient computational strategies based on single-reference quantum-chemistry methods have been developed enabling determinations of line shapes or, in some cases, achieving rovibrational resolution. Developments are however lacking for strongly correlated problems, with many excited states, high-order excitations, and/or near degeneracies between states of the same and different spin multiplicities. In this work, we define and compare the performance of distinct computational strategies using multiconfigurational quantum chemistry, nuclear sampling of the chromophore (by means of molecular dynamics, ab initio molecular dynamics, or Wigner sampling), and conformational and statistical sampling of the environment (by means of molecular dynamics). A new mathematical approach revisiting previous absolute orientation algorithms is also developed to improve alignments of geometries. These approaches are benchmarked through the *n*π* band of acrolein not only in the gas phase and water solution but also in a gas-phase/water interface, a common situation for instance in atmospheric chemistry. Subsequently, the best strategy is used to compute the absorption band for the adduct formed upon addition of an OH radical to the C6 position of uracil and compared with the available experimental data. Overall, quantum Wigner sampling of the chromophore with molecular dynamics sampling of the environment with CASPT2 electronic-structure determinations arise as a powerful methodology to predict meaningful σ(λ) and ε(λ) band line shapes with accurate absolute intensities.

## Introduction

Theoretical molecular electronic spectroscopy is based on the characterization of the electronic structure of several electronic states and the determination of the electronic transition energies and intensities. Since the early studies in this field, a first approach to obtain the absorption electronic spectra has been the determination of the ground state equilibrium structure of the isolated molecule and next the computation, at this geometry, of the vertical transition energies (*E*_va_^0→*l*^) from the ground (0) to several excited (*l*) states and the oscillator strength for each transition (*f*_0→*n*_) via the transition dipole moment (μ⃗_0→*l*_) and the formula *f*_0→*l*_ = 2/3 *E*_va_^0→*l*^|μ⃗_0→*l*_|^2^, in atomic units.^1^ This approach is sometimes good enough to solve certain chemical problems induced by light absorption,^2^ especially in combination with accurate multiconfigurational methods such as the complete-active-space self-consistent field second-order perturbation theory (CASPT2).^1,3,4^ However, in many situations, a more accurate description is required, and it is needed to go beyond this single-geometry approximation, for example, when vibrations strongly affect the absorption properties^5^ or when photochemical rates (*J*) are required to build kinetic models of the physical and chemical processes induced by light.^6−10^ For the latter, absorption intensities (cross sections (σ)) over the wavelength (σ(λ)) must be computed, *J* = ∫ ϕ(λ) σ(λ) *I*(λ) dλ being the formula for the photochemical rate *J*, where ϕ(λ) and *I*(λ) are the quantum yield of the process and the irradiance of the light source, respectively.

An optimal approach to address the mentioned cases (and others) is to obtain the rovibrational structure of the electronic bands. In this context, some interesting methodologies have been developed. One of them is to compute, within quantum dynamics, the autocorrelation function *C*(*t*) = ⟨Ψ(0)|Ψ(*t*)⟩, where Ψ(0) represents the ground-state wave function promoted instantaneously on the manifold of the excited state and Ψ(*t*) refers to the evolved wave function at time *t*. *C*(*t*) is related to σ(λ) by means of the expression σ(ω) ∝ ω ∫_–∞_^∞^ d*tC*(*t*) e^*iωt*^, where the energetic dependence on the wave numbers ω (ω = 2π*c*/λ, *c* being the speed of light) is shown.^11^ Another useful approach, which also follows a quantum approach, this time with a static (time-independent) procedure, is the normal modes approach. Here, the equilibrium structure of the ground and excited electronic states is obtained, the vibrational states are approximated by means of the Hessian matrices, and harmonic models and rovibronic couplings are thereby computed. This strategy has been commonly applied with time-dependent density functional theory (TDDFT),^12−15^ even though studies with complete-active-space self-consistent field (CASSCF) and CASPT2 and coupled-cluster methods have also been reported (see, for example, refs (16−19)). Note that the vibronic spectra can be also computed in the time-domain rather than in the frequency domain in an efficient manner as shown by Tapavicza and co-workers.^20,21^

The two mentioned approaches allow an accurate resolution of the electronic bands. However, they are limited to a low number of excited states with relatively low complexity. Quantum dynamics on several states becomes very computationally demanding, and the approximations in the normal mode approach with TDDFT become weak in strongly correlated problems, with many degeneracies between states of the same and distinct spin multiplicities and electronic configurations implying high-order excitations.^3,22,23^

A further step to improve spectroscopic determinations with computational chemistry is to consider the chromophore embedded into real environments (solutions, interfaces or surfaces, protein or nucleic acid environments, etc.) Apart from the widely employed reaction field models,^24^ molecular dynamics (MD) or Monte Carlo simulations using classical mechanics often provide good accuracy for a conformational sampling of the macromolecular system surrounding the chromophore. The part of the system at which the excitation is localized in the absorption process must be treated with quantum chemistry methods due to the quantum nature of the electronic excitation phenomenon. For computing the interactions between the chromophore and the atoms from the environment, additive and subtractive quantum mechanics/molecular mechanics (QM/MM) schemes,^25,26^ averaged solvent electrostatic potential (ASEP),^27^ density embedding,^28,29^ etc. have been implemented in common quantum-chemistry packages of software.

Static and dynamic computational strategies with distinct combinations of MD, QM, and QM/MM schemes (with two or more layers) have been extensively used in the literature to study fully solvated chromophores, usually disentangling the different solvent-chromophore contributions or improving the models that describe the light–matter interaction in these complex systems.^15,30−42^ Nuclear quantum effects have been shown to be relevant to correctly capture the general features of the line shape in the absorption spectra.^43−49^ In this context, ab initio path integral MD (PIMD) arises as an accurate technique for the inclusion of such quantum effects, considering also the anharmonicity of the molecular system, even though at a high computational cost. So far, these studies provide efficient approaches for addressing single-reference problems.

In this work we aim at complementing the previous studies with an efficient and practical computational protocol for determining molar extinction coefficients (ε(λ)) in condensed phases for strongly electron correlated problems, that is, situations with a large number of excited electronic states (with both mono- and multiconfigurational character), near-degeneracies between states of the same and distinct spin multiplicity, complex open-shell systems, etc. The approach must also be able to predict band line shapes (ε(λ)), accounting for the environmental (statistical and conformational) effects on the electronic structure. Such tools allowing predictions of absolute values of ε(λ) with the accuracy of multireference quantum chemistry methodologies shall be valuable, among other fields, in atmospheric chemistry to extend determinations of photolysis rates (*J*) from the gas phase^8,9,50^ to water droplets, ice, or aerosols.

For that, the performance of distinct strategies for modeling the macromolecular system is compared. In all of them, we follow a procedure in which, first, a representative ensemble of nuclei geometries is obtained, next, vertical excitation energies (*E*_*va*_^0→*l*^) and oscillator strengths (*f*_0→*l*_) for several excited states (*l*) are computed for all the structures, and finally, the data are convoluted. We also develop and apply a novel mathematical method based on the polar decomposition to introduce structures into solvent cavities ensuring the best fitting of the chromophore inside the mentioned cavity, thus providing optimal solvent–solute interactions for an efficient computation of the effects of the environment on the optical properties of the embedded chromophore.

Note that the procedure used herein to compute the spectra does not allow a rovibrational resolution included in the Franck–Condon and correlation function based approaches, due to the lack of vibronic transitions (the high-energy vibronic tail will not be captured).^51^ Contrarily, we focus on the general estimation of the line shape and absolute values of σ(λ) and ε(λ).

**Chart 1:**
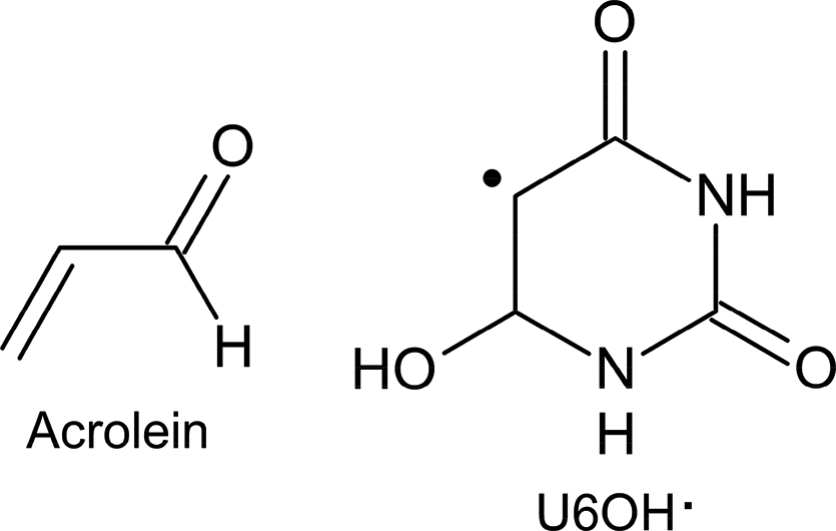
Chemical Structures of Acrolein and U6OH^•^

The distinct strategies are first benchmarked in a well-known organic molecule, acrolein (see Chart 1). The lowest-lying excited state of this molecule is an *n*π* transition, which is blue-shifted from gas phase to an aqueous medium.^52−59^ This shift is well-defined in the experiments and has been used in many studies to test new developments and implementations of methodologies to treat solvation effects.^60−65^ For similar systems as acrolein in air–water interfaces, Ruiz-López and co-workers showed significant changes in the absorption spectrum with respect to that computed in the gas phase.^66^ Second, the best performing multiconfigurational quantum-chemistry computational approach is herein applied to correctly ascribe the spectrum in the visible range measured experimentally after the reaction of OH radicals with uracil,^67^ attributing the transient absorption band to the adduct at the C6 position (U6OH^•^; see Chart 1). This clearly confirms previous estimations on the isolated molecule that did not consider the nuclear sampling.^68^ Note here that on the basis of single-reference methods, in particular, TDDFT, the experimental absorption band was incorrectly assigned to the U5OH radical.^69^

## Methodology

The computational strategies compared herein to determine σ(λ)/ε(λ) with multiconfigurational quantum chemistry have the following structure:

(1) *Chromophore nuclear sampling (for σ and ε) and solvent conformational and statistical sampling (only for ε)*. With this, a significant number of nuclear positions of the solvent and chromophore, which are representative of the vibrations of the atoms at standard conditions, were generated.

(2) *Determination of E*_*va*_^*0*→*l*^*and f*_*0*→*l*_. From the geometries obtained in 1, QM (for σ) and QM/MM (for ε) methodologies were used to determine a set of *E*_va_^0→*l*^ and associated *f*_0→*l*_.

(3) *σ, ε values.* The computed *E*_va_^0→*l*^ and *f*_0→*l*_ were combined to obtain σ or ε as a function of the wavelength (λ) of the incident radiation.

The computational details used for each point are indicated in the following subsections.

### Chromophore Nuclear Sampling and Solvent Conformational and Statistical Sampling

Three distinct strategies were used to generate the ensemble of geometries of the chromophore in the gas phase: (i) *molecular (classical) dynamics (MD)*, with parametrized molecular mechanics (MM) force fields; (ii) *ab initio molecular dynamics (AIMD)*, in which quantum chemistry was used for describing the electrons of the chromophore and the nuclei moved according to classical equations; and (iii) *Wigner sampling (WS)*, where frequencies were computed at the equilibrium ground state structure with quantum chemistry and a Wigner distribution was generated.^70^ Strategies i, ii, and iii were used for acrolein, while only strategy iii was applied to the U6OH radical. For the chromophore-solvent macromolecular systems, solvent geometries were sampled with MD simulations, giving rise therefore to the following three strategies, which are analogous to those for the gas phase, (i) *MD*; (ii) *QM/MM MD*, where the chromophore is described at the QM level and the solvent with MM; and (iii) *WS+MD*, where the chromophore is sampled with WS and the solvent with MD. The classical MD sampling used herein is related to the approach used by García-Iriepa, Navizet, and co-workers,^71−73^ whereas the Wigner sampling is based on a previous approach defined and used for gas phase computations (see our previous works refs (7−10, 74)) and can be considered an adaptation from single-reference methods to multiconfigurational quantum chemistry of the protocols by Crespo-Otero and Barbatti,^75^ for the gas phase, and by Lischka and co-workers,^76−78^ for condensed phases. As for the gas phase, i–iii are used in acrolein and iii in the U6OH radical.

#### Classical MD (Gas Phase) Sampling

Under isothermal–isobaric ensemble (NPT) conditions (300 K and 1 bar), a 2 ns classical MD simulation with periodic boundary conditions was performed with a 2 fs time step in the gas phase with the Amber14 program,^79,80^ analogously as for solvent conditions (see below). During these simulations, the pressure and temperature were maintained constant by using the Berendsen algorithm, and the SHAKE algorithm was used to restrain the movement of the C–H bonds. The parameters set for acrolein (equilibrium bond lengths and equilibrium angles) were obtained from a DFT geometry optimization with the B3LYP functional^81,82^ and the 6-311G(2d,p) basis set using the Gaussian 09 D.01 program package.^83^ The point charges of the parameters set for acrolein were taken from the Mulliken charges of the DFT optimization. The force constants were taken from the GAFF force field.^84^ After the simulation, an ensemble of 100 structures was taken from the equilibrated dynamics uniformly separated by 20 ps for the absorption spectra determinations.

#### Classical MD (Condensed Phase) Sampling

Two nanosecond NPT MD simulations were done with Amber14 at 300 K and 1 bar, with periodic boundary conditions and a 2 fs time step, using the Berendsen algorithm to maintain constant the temperature and pressure and employing the SHAKE algorithm to restrain the movement of the C–H bonds. The TIP3P force field^85^ was used for the water molecules, and for acrolein, the parameters were obtained as follows. First, a MD simulation was performed with the parameters obtained from the gas phase B3LYP/6-311G(2d,p) geometry optimization. Next, the lowest-in-energy snapshot was extracted, and acrolein was further optimized in the ground state at the B3LYP/6-311G(2d,p) level of theory with a hybrid QM/MM scheme in solution, with Gaussian09+Tinker.^83,86^ A new set of parameters (equilibrium bond lengths, angles, and Mulliken charges) was then taken from this QM/MM optimization, and a new 2 ns MD simulation with this new parameter set was performed. Two ensembles of 100 snapshots were taken by extracting one snapshot every 20 ps from each of the two MD simulations carried out. Subsequently, QM/MM vertical transitions (see Determination of *E*_va_^0→*l*^ and *f*_0→*l*_) were calculated to simulate the absorption spectra from the two ensembles. As the spectral shapes were similar and the difference of energy of the maximum of the two simulated absorption spectra was less than 0.2 eV, no further iterations were done. As the sampling depends on the parameter set used for the dynamics, this iterative procedure of determining the parameters can be necessary in some cases (see refs (71−73)). In the case of acrolein, the first iteration was enough. After finishing the dynamics simulations, an ensemble of 100 structures was taken from the last equilibrated dynamics uniformly separated by 20 ps for the absorption spectra computations.

#### AIMD (Gas Phase) Sampling

An AIMD simulation of 10 ps was carried out from one snapshot extracted (the lowest in energy) from the classical 2 ns MD simulation in the gas phase. The simulation was done using the Sander program of Amber14 (for the propagator algorithm) calling Gaussian 16^87^ (for the computations of the energies).^88^ The temperature was set to 300 K using the Langevin thermostat and the pressure to 1 bar. The time step was set to 1 fs. The acrolein atoms were specified to be treated at the B3LYP/6-31G level. Once the simulation was done, 100 snapshots were selected for the spectra determinations (one snapshot every 0.1 ps from the AIMD simulation).

#### QM/MM MD (Condensed Phase)

A series of QM/MM MD simulations of 1 ps were performed from 10 snapshots selected uniformly separated (taken every 200 ps) from the classical 2 ns MD simulations. As for the AIMD simulations in the gas phase, the Sander program of the Amber14 suite of programs was used coupled with Gaussian 16. The temperature was set to 300 K using the Langevin thermostat and the pressure to 1 bar. The time step set of 1 fs was used. The acrolein molecule was described at the B3LYP/6-31G level and the water molecules treated classically with the TIP3P force field. As the computational demand for QM/MM is higher than for classical MD, the dynamics were shorter. A total of 100 snapshots were selected for the spectra computations (one snapshot every 0.1 ps from each of the 10 QM/MM MD simulations).

#### WS (Gas Phase) Sampling

In the gas phase, a set of 100 geometries was obtained according to a Wigner distribution^70^ for the optimized geometry and using the vibrational harmonic frequencies of the normal modes obtained at the DFT/B3LYP/6-31G and DFT/B3LYP/6-311G(2d,p) levels, for acrolein, and at the DFT/M06-2X/6-31++G(d,p) level, for the U6OH radical (as benchmarked elsewhere),^68,89^ with Gaussian 09.

#### WS+MD (Condensed Phase) Sampling

**Figure 1:**
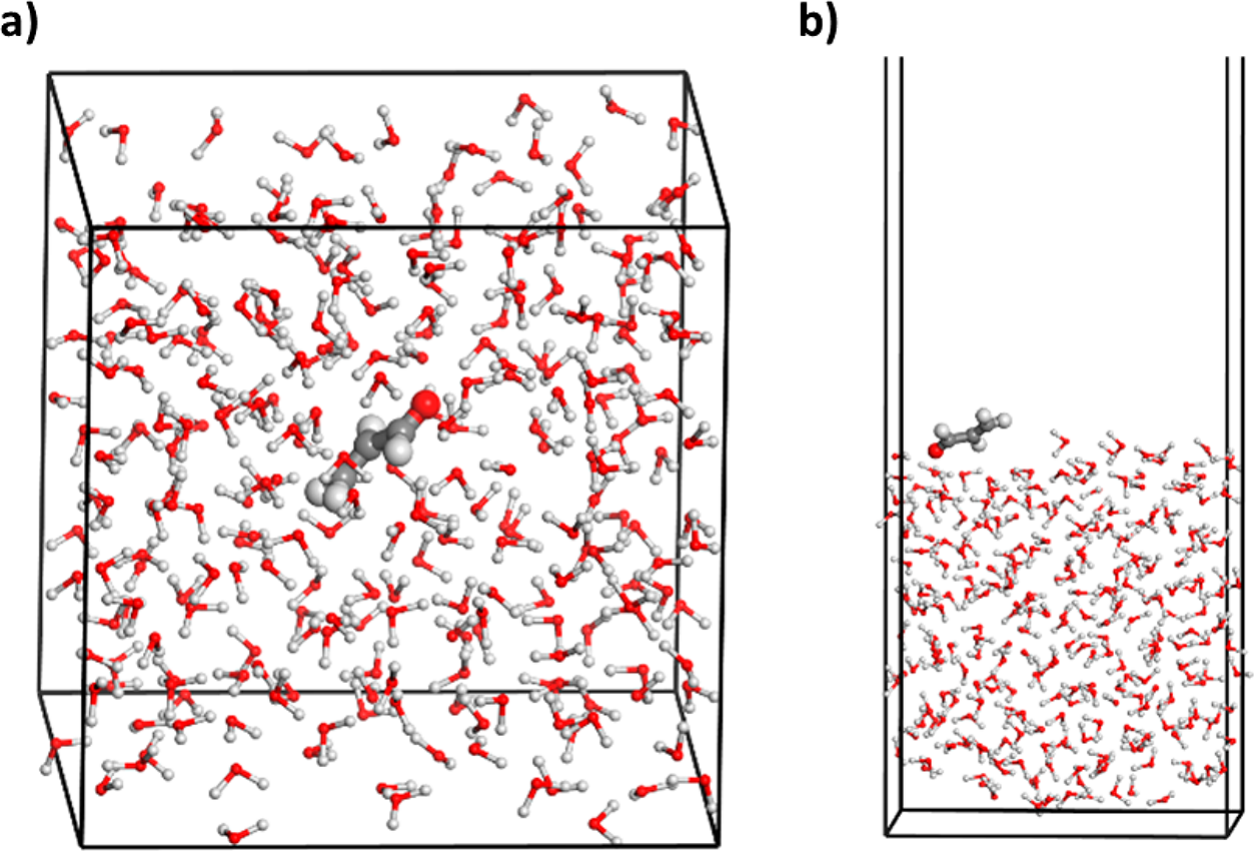
(a) Snapshot of the WS+MD simulations performed for acrolein in water solution generated with the Materials Studio software.^90^ (b) Representative snapshot at the gas phase–water interface with the analogous WS+MD approach (see text).

In water solution, first, QM computations at the DFT/B3LYP/6-31G and DFT/M06-2X/6-31++G(d,p) levels of theory and using the polarizable implicit model (PCM)^24^ with Gaussian 09 were performed to obtain the optimized geometry and frequencies of acrolein with two and nine explicit water molecules for acrolein and U6OH^•^, respectively (forming hydrogen bonds with the carbonyl, amino, and alcohol groups). Second, similarly as in the gas phase, a set of 100 structures was generated according to a Wigner distribution. Third, two independent MD simulations, one with Amber14 and the other with the Materials Studio 2019 package of software,^90^ were run in acrolein to compare slightly different implementations; for U6OH^•^, only the latter was used. With Amber14, an MD simulation of 1 ns of acrolein in a box of water was performed for a fixed geometry of the solute using the DFT equilibrium structure and electrostatic potential fitted (ESP)^91,92^ charges for the atoms of acrolein computed at the same QM level of theory (removing the two explicit water molecules). TIP3P parameters were used for the water molecules. To fix the geometry of acrolein, we tested two strategies either increasing the masses of the atoms by 10^7^ amu or adding a restraint constant of 10^5^ kcal mol^–1^ Å^–2^ to their Cartesian coordinates, with the same outcome. For Materials Studio 2019, an MD simulation of 1 ns with NVT conditions at 300 K and 1 bar with the Nosé thermostat^93−95^ and a time step of 1 fs was then performed with the Forcite module of the Materials Studio 2019 package of software^90^ and the fixed acrolein or U6OH^•^ geometry optimized with DFT. Cartesian coordinates were constrained here to be fixed, observing the same behavior as in Amber14. The Amorphous Cell module of the program was initially used, which uses MM computations to efficiently produce appropriate conditions for the subsequent dynamics. The general Dreiding force field^96^ with simple-point charges (SPC)^97,98^ was used for water and ESP charges for the acrolein and U6OH^•^. Fourth, 10 snapshots were selected from the MD simulations of Amber14 in the production region, and the acrolein geometries were replaced randomly with those obtained with the 100 Wigner distribution (10 per each, ratio of 100-in-10). For this replacement, a program was herein developed (*lstrans* program) with a modified mathematical approach to solve the problem of geometry alignments. This approach is based on the polar decomposition (see Golub and van Loan),^99^ follows a least-squares fitting of the atom positions, and ensures that the solution found in the alignment is a minimum, in contrast to other mathematical procedures available in the literature that only provide stationary points (the mathematical basis and proofs of the *lstrans* program can be found in the SI).^100−102^ For the MD simulations of Materials Studio, 25 snapshots were taken (in acrolein and U6OH^•^) and replaced similarly by the 100 Wigner geometries (four per each, ratio of 100-in-25). The water molecules were kept at the same positions in such procedures. Fifth, for each of the two MD simulations (with Amber14 and Materials Studio 2019), 100 snapshots were extracted, and for each one of the snapshots, classical MD simulations of 10 ps with Amber14 or of 5 ps with Materials Studio were performed while freezing the chromophore Wigner geometry as described above to allow the water molecules to adapt to the new geometry of the acrolein or U6OH^•^. Finally, the last snapshot of these short-time MD simulations was chosen leading to two ensembles of 100 snapshots, one from the Amber14 MD and one from the Materials Studio MD, for determining the respective absorption spectra. See a representative snapshot for the Materials Studio simulations in water solution in Figure 1a.

Under the water surface conditions (only for acrolein), a 1 ns MD simulation of a water–gas interface was carried out (without acrolein) with Materials Studio 2019. Next, five snapshots uniformly separated were extracted, and a simulated annealing procedure using Monte Carlo with the Adsorption Locator module of Materials Studio was followed in each one to obtain the five most stable adsorption interactions between acrolein and the surface water molecules. This gave a total of 25 structures that were replaced by the 100 Wigner geometries (four per each, randomly, ratio of 100-in-25) with the *lstrans* program, similarly to the water solution computations described above. Five picosecond classical MD simulations with the Materials Studio program were also performed for freezing the acrolein Wigner geometry, giving rise to a total amount of 100 sampled geometries taken from the last snapshot of the MD computations. See a representative snapshot for the Materials Studio simulations at the water–air interface in Figure 1b.

### Determination of *E*_va_^0→*l*^ and *f*_0→*l*_

QM and QM/MM electrostatic embedding computations were carried out with the OpenMolcas program^103^ for the gas phase and condensed phases, respectively, with the CASSCF/CASPT2 method for the chromophore and using the same field of charges for the solvent atoms as in the corresponding MD simulations performed in the previous step to generate the nuclear sampling.

For acrolein, the active space was composed by six active electrons distributed in five active orbitals (hereafter, CAS(6,5)), including the valence π, π* and oxygen lone pair (*n*) orbitals. For the ^•^OH adduct of uracil at the C6 position, i.e., U6OH^•^, there was an active space of 15 active electrons distributed into 10 active orbitals (all the π bonding and π* antibonding orbitals plus the oxygen and nitrogen lone-pair orbitals of the C=O, =N–, and −OH groups; hereafter, CAS(15,10)). In the CASSCF computations, a state average procedure of eight and 10 roots was used for obtaining the wave functions of acrolein and U6OH^•^, respectively. The atomic natural orbitals large-type basis sets contracted to C, N, O [4s3p2d1f]/H [3s2p1d] (ANO-L-VTZP) and to C, N, O [3s,2p,1d]/H [2s1p] (ANO-S-VDZP) were used for acrolein and U6OH^•^, respectively.^104,105^

The dynamic electron correlation was subsequently computed on top of the state-average CASSCF wave functions by means of the CASPT2 method within the state-specific approach. An imaginary shift^106^ of 0.2 au was used to minimize the effect of intruder states, and the IPEA shift^107^ was set to 0.0 and 0.25 au, for U6OH^•^ and acrolein, respectively, to maintain the consistency with respect to our previous works on acrolein^63^ and U6OH^•^.^68,89^

### σ(λ) and ε(λ) Determinations

The computed *E*_va_^0→*l*^ and *f*_0→*l*_ were combined to produce σ in cm^2^ using the following formula:
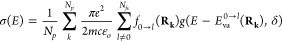
where *e* and *m* are the charge and mass of the electron, respectively, *c* is the speed of light in a vacuum, ε_*o*_ is the vacuum permittivity, *N*_*p*_ is the number of sampled geometries (100), *N*_*fs*_ is the number of excited states, *f*_0→*l*_ is the oscillator strength of the transition from the ground state to the *l*th excited state calculated separately for each geometry **R**_**k**_, and *g*(*E* – *E*_va_^0→*l*^(**R**_**k**_),δ) is the Gaussian-type shape function that accounts for the broadening of the resonant lines of the spectra:
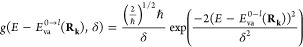
Each of the Gaussian functions is centered at the vertical transition energy, *E*_va_^0→*l*^(**R**_**k**_), and δ is the phenomenological broadening (0.2 eV). Such a δ term is needed to convert all the discrete computed pairs of *E*_va_^0→*l*^ and *f*_0→*l*_ into lineshapes. Since the computational strategies that we used herein are not able to predict the rovibrational structure of the electronic bands, we chose the smallest phenomenological broadening value that does not show unphysical peaks resembling such rovibrational resolution.

The statistical error of the sampling (δσ(*E*)) was measured as the standard deviation for the particular sampled photon energy *E*:
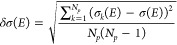
where σ_*k*_(*E*) is the signal at photon energy *E* obtained from a particular geometry **R**_**k**_:
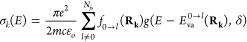
ε(*E*) and δε(*E*) in M^–1^ cm^–1^ were obtained with the following formula to convert the units:
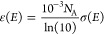

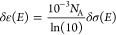
where *N*_A_ is the Avogadro constant.

Note that δσ(*E*) and δε(*E*) are random errors caused by representing the whole population only with a subset. For further details on the source of this type of error and the characteristics, we refer to the work of Srsen et al.^46^

## Results and Discussion

### Acrolein: Gas Phase, Water Solution, and Water–Gas Interface

**Figure 2:**
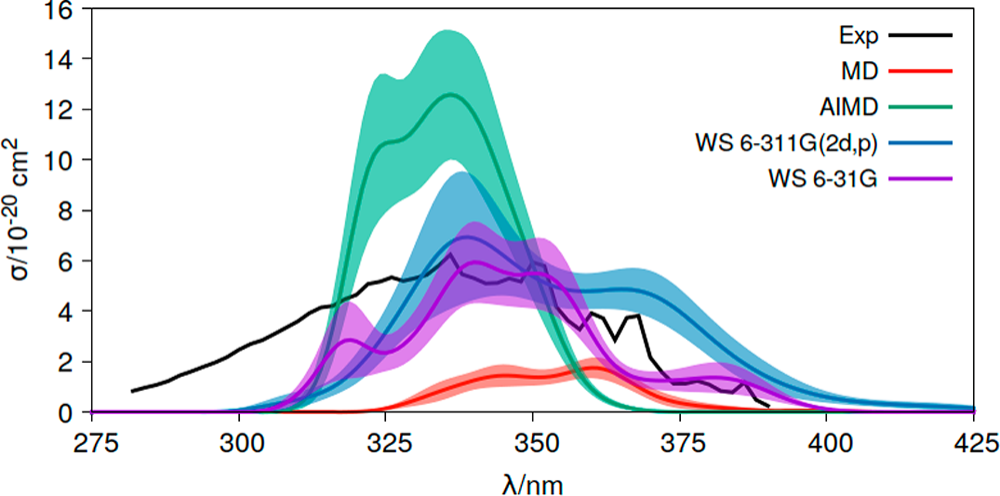
Absorption cross sections (σ) of acrolein in the gas phase measured experimentally (Exp)^55,57^ and computed at the CASPT2(6,5)/ANO-L-VTZP level of theory using distinct conformational sampling approaches, classical molecular dynamics (MD), ab initio molecular dynamics (AIMD), and Wigner sampling (WS). For WS, two basis sets are compared, 6-31G (WS 6-31G) and 6-311G(2d,p) (WS 6-311G(2d,p)). Shadow areas represent the statistical error of the approach (δσ(*E*)).

The lowest-lying excited state of acrolein mainly corresponds to an electron excitation from the oxygen lone pair orbital of carbonyl to an antibonding π* orbital (*n*π* electronic transition). The *E*_va_^0→*l*^ is found to be 3.66 eV (339 nm) and 3.52 eV (352 nm) at the CASPT2/ANO-L-VTZP//B3LYP/6-31G and CASPT2/ANO-L-VTZP//B3LYP/6-311G(2d,p) levels of theory, respectively. The *f*_0→*l*_ is negligible for this transition at planar geometries. Figure 2 shows the σ(λ) results computed for acrolein in the gas phase only for the *n*π* electronic transition (related to the first excited state) using the classical MD, AIMD, and WS computational strategies described in the previous section and the band line measured experimentally for the same conditions.^55,57^ Overall, the three approaches agree reasonably well regarding the energy region of the absorption band, and the intensities appear within the same order of magnitude in general. Nevertheless, two main general qualitative differences can be observed in the comparison between the sampling approaches. First, MD and AIMD show a low broadening of the band, whereas the WS approach provides a broader band, thus approaching the experimental record. This difference in the broadening has been identified also in other studies^34^ and can be attributed to the lack of nuclear quantum effects (zero-point vibrational energy (ZPVE)) of the acrolein geometries obtained in MD and AIMD.^44−48^ The classical treatment of the vibrations in these two approaches allows less stretched internal coordinates as compared to those obtained by WS (see Figures S1–S3). To analyze possible unphysical effects of the phenomenological broadening δ parameter, we have reconvoluted the bands, drastically reducing the δ value to 0.001. The normalized intensities (Figure S4) show in general a wider dispersion of peaks for the WS ensemble, confirming that there is a physical reason for the enhanced broadening in this approach as compared to the others apart from the broadening that could arise from the δ parameter. The second observation that can be made from Figure 2 is that AIMD overestimates the intensities, while classical MD underestimates them, and WS significantly improves this description. Test computations increasing the basis set used to obtain the frequencies within the WS approach from 6-31G to 6-311G(2d,p) maintain the general description and the differences with respect to the MD and AIMD data. Therefore, the basis set does not introduce a significantly high change in this aspect. Analysis of the distribution of the dihedral angle formed by the heavy atoms (O=C–C=C; Figure S5) does help to interpret this result. Thus, a correlation exists between the spread of dihedral angles and the absorption intensities. AIMD produces the highest out-of-plane distortions, which enhances the oscillator strength of the *n*π* electronic transition and gives rise to more intense bands in acrolein. MD samples a short range of dihedral angles located at 180°, that is, almost planar geometries, and accordingly, transitions are less intense. Note finally that the lack of vibronic couplings in the three approaches theoretically fails to describe correctly the origin in the spectra (if the Franck–Condon term is exactly zero in the Herzberg–Teller approximation, as is the case for the *n*π* state of acrolein, the first allowed transition requires a quantum on the vibrational mode that induces an intensity borrowed from other states). Nevertheless, this effect is very likely to be overwhelmed by the width of the spectrum.

For condensed phases (water solution and gas phase–water interface), Figure 1a shows a representative structure of the system from the dynamics simulations. In Figure S6, the organization of water around acrolein in the classical MD and WS approaches is compared, using as comparative parameters the associated radial distribution function between the atoms of acrolein and those of the water molecules (*g*(*r*)). As described above in the Methodology section, in WS+MD, a 1 ns classical MD simulation with a fixed chromophore was first carried out, extracting 10 snapshots from the production zone uniformly separated to have 10 different and representative solvent arrangements. Then, 100 geometries from the Wigner distribution of acrolein were randomly inserted into the 10 snapshots (10 Wigner structures in each of the snapshots, in total 100-in-10) and relaxed with 10 ps MD keeping the geometrical parameters of the chromophore frozen (*g*(*r*) values in Figure S6, red curve). For the sake of comparisons, 100 snapshots from the 1 ns MD were extracted and analyzed (*g*(*r*) values in Figure S6, blue curve). Also, the 100 Wigner geometries were inserted into the 100 snapshots from the 1 ns MD simulation (100-in-100 ratio), and the obtained structures were also relaxed with 10 ps MD with frozen acrolein geometry (*g*(*r*) values in Figure S6, green curve). Note that *g*(*r*) values are almost identical for the three cases, which validates the relaxation protocol and ensures a complete statistical sampling of the solvent around acrolein.

**Figure 3:**
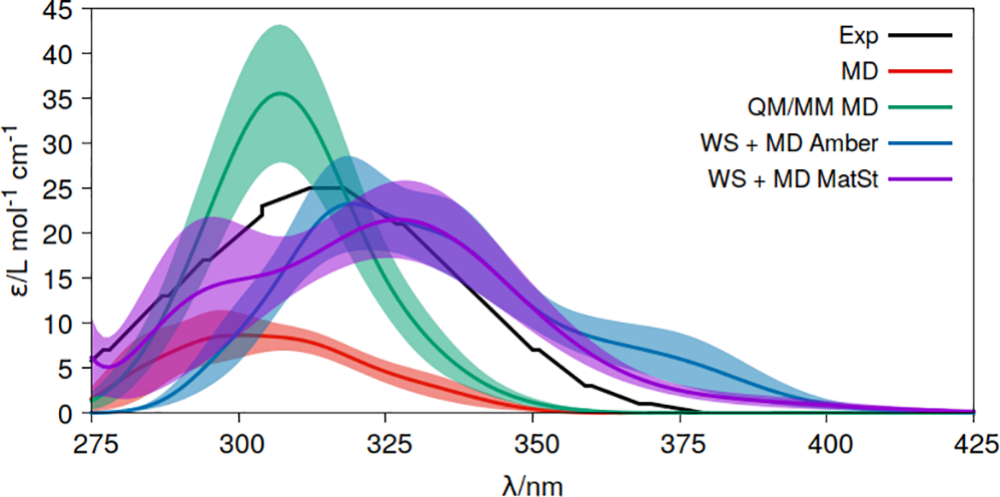
Molar extinction coefficients (ε) of acrolein in water solution measured experimentally (Exp)^59^ and computed at the CASPT2(6,5)/ANO-L-VTZP level of theory using distinct conformational sampling approaches, classical molecular dynamics (MD), quantum mechanics/molecular mechanics molecular dynamics (QM/MM MD), and Wigner and molecular dynamics sampling for the chromophore and solvent, respectively (WS+MD). For WS+MD, two implementations are compared, Amber (WS+MD Amber) and Materials Studio (WS+MD MatSt). Shadow areas represent the statistical error of the approach (δε(*E*)).

The ε values obtained for water solution with MD, QM/MM MD, and WS+MD are displayed in Figure 3. Similar trends as those discussed for the gas phase are found. Thus, the WS+MD band is broader than those obtained at the MD and QM/MM MD levels, reaching a closer broadness to the experimental one.^59^ This can be attributed to the broader distribution of bond lengths generated when considering the nuclear quantum effects with the WS+MD approach, as shown in Figures S7–S9. The relative broadening is also maintained here when the convolution is done with δ = 0.001 (see Figure S10), discarding unphysical effects of the δ parameter. The band height for WS+MD is also found in between that of MD and QM/MM MD in correlation with the amount of out-of-plane distorted geometries from the nuclear ensemble of the chromophore (Figure S11). Despite the distinct ratios between Wigner geometries and MD conformations in Amber14 and Materials Studio (100-in-10 and 100-in-25, respectively), their bands differ less than with respect to the curves obtained with MD and QM/MM MD. Moreover, it is worth noting here that, within the WS+MD approach, the different force fields and implementations used with either Materials Studio or Amber for obtaining the conformational and statistical sampling of the solvent give similar results. An advantage of the Materials Studio program is that it has better optimized tools and a practical and efficient interface, which facilitates the preparation of the system and the application of the WS+MD strategy.

Another aspect that is worth mentioning at this point is the anharmonicity description by the distinct sampling approaches. Thermal sampling used in MD and AIMD allow capturing it. Meanwhile, pure WS uses harmonic frequencies and therefore lacks an anharmonic treatment of the vibrations used to generate the sampling.^108^ Nevertheless, anharmonicity becomes relevant for the low frequencies, which are especially significant in condensed phases and are mainly related to the degrees of freedom of the nearby solvent environment. In this sense, WS+MD captures that anharmonicity part, lacking only that related to the low frequencies of the chromophore. Despite this deficiency of WS+MD, it can be seen in Figure 3 that deviations from the experimental band are lower for this approach as compared to the others. This indicates that nuclear quantum effects are likely more important than anharmonicity to correctly sample the conformational space of the chromophores. In any case, modification of the WS to incorporate anharmonic corrections with PIMD^48^ or ideally with less computationally demanding techniques to compensate for the efforts of multiconfigurational quantum chemistry required for strongly correlated problems could be addressed in future studies to further improve the description.

**Figure 4:**
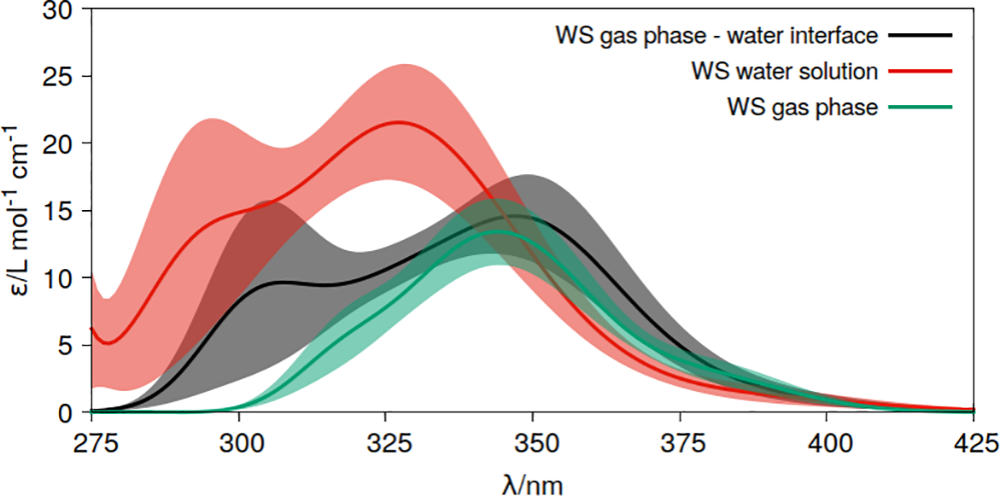
Molar extinction coefficients (ε) of acrolein in distinct media, gas phase, water solution and at the gas phase–water interface computed at the CASPT2(6,5)/ANO-L-VTZP level of theory using the Wigner sampling (WS) strategy. Shadow areas represent the statistical error of the approach (δε(*E*)).

The aforementioned practical advantages of Materials Studio are even more useful when we aim at determining ε at the gas phase–water interfaces (Figure 1b). Figure 4 shows the corresponding results in these conditions, comparing the data with that obtained for the gas phase and water solution. The blue shift of the *n*π* band measured experimentally for acrolein absorption from the gas phase to water solution is clear. Note also the increase of intensities also reproducing the experimental trend.^55,57,59^ For the gas phase–water interface, it maintains the gas phase electronic band signals. These arise from the acrolein molecules that are surrounded by the side without solvent. In addition, a shoulder at shorter wavelengths (higher energies) is obtained, which can be attributed to the acrolein structures interacting more strongly with the side of the system containing water.

### Uracil OH Radical: Gas Phase and Aqueous Solution

As described in the Introduction, TDDFT vertical determinations based solely on the ground-state equilibrium geometry gave rise in previous works^69^ to a wrong identification of the radical responsible for the visible absorption band appearing in the experimental spectrum. This is due to the fact that the same energy absorption was obtained for U6OH^•^ (396 nm) and U5OH^•^ (387 nm) at the ground-state equilibrium structures.^69^ On the contrary, CASPT2 data computed at the optimized geometry of the ground-state determined an energy of 406 nm for U6OH^•^, far enough from the other species (282 nm).^68^

**Figure 5:**
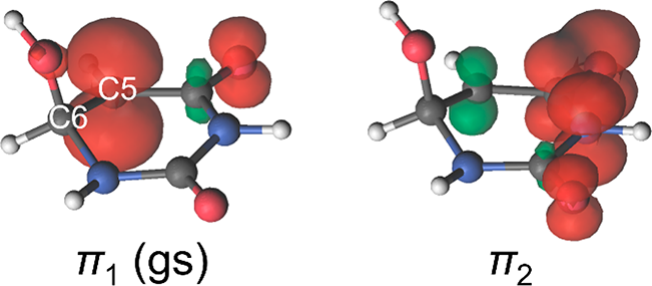
Spin density (unpaired electron) plots for the π_1_ (D_1_) and π_2_ (D_3_) states of U6OH^•^. Reproduced with permission from ref (68). Copyright 2013 American Institute of Physics.

The electronic excitations of U6OH^•^ are characterized by redistributions of the spin density from the C5 atom (π_1_, D_1_ ground state) to the carbonyl group (n_O_ states) or π-like orbitals delocalized over the ring (π_2_, π_3_, ... states).^68,89^ The latter have the largest oscillator strengths and thus dominate the absorption processes. At visible wavelengths, the most intense absorption is the π_1_ (D_1_) → π_2_ (D_3_) transition (Table 1), which is illustrated by the corresponding spin density of the states plotted in Figure 5.

**Table 1:** Vertical Absorption Energies (*E*_va_^0→*l*^) and Wavelengths (λ_va_^0→*l*^) and Oscillator Strengths (*f*_0→*l*_) of the First Nine Doublet Excited (D_2_-D_10_) States of U6OH^•^ Computed with the CASPT2(15,10)/ANO-S-VDZP Methodology, at the M06-2X/6-31++G(d,p) Ground State (D_1_) Equilibrium Geometry in the Gas Phase

state	*E*_va_^0→*l*^ (eV)	λ_va_^0→*l*^ (nm)	*f*_0→*l*_
D_2_	2.29	541	0.0002
D_3_	2.90	428	0.0238
D_4_	3.91	317	0.0020
D_5_	3.96	313	0.0053
D_6_	4.51	275	0.0027
D_7_	4.74	262	0.0022
D_8_	5.70	217	0.0150
D_9_	6.03	206	0.0004
D_10_	6.04	205	0.0004

We applied at this point the best performing approach for chromophore-environment sampling found in acrolein (WS+MD) to determine the absorption spectra of U6OH^•^ using the computational details described in the Methodology section based on the Materials Studio 2019 program. The 9 electronic transitions listed in Table 1 were considered for the simulation. The spectrum produced was compared with the experimental spectrum in the visible wavelength range (band maximum peaking at ∼400 nm) measured in an aqueous solution of uracil in which OH uracil radicals were generated by radiolysis of water.^67^

**Figure 6:**
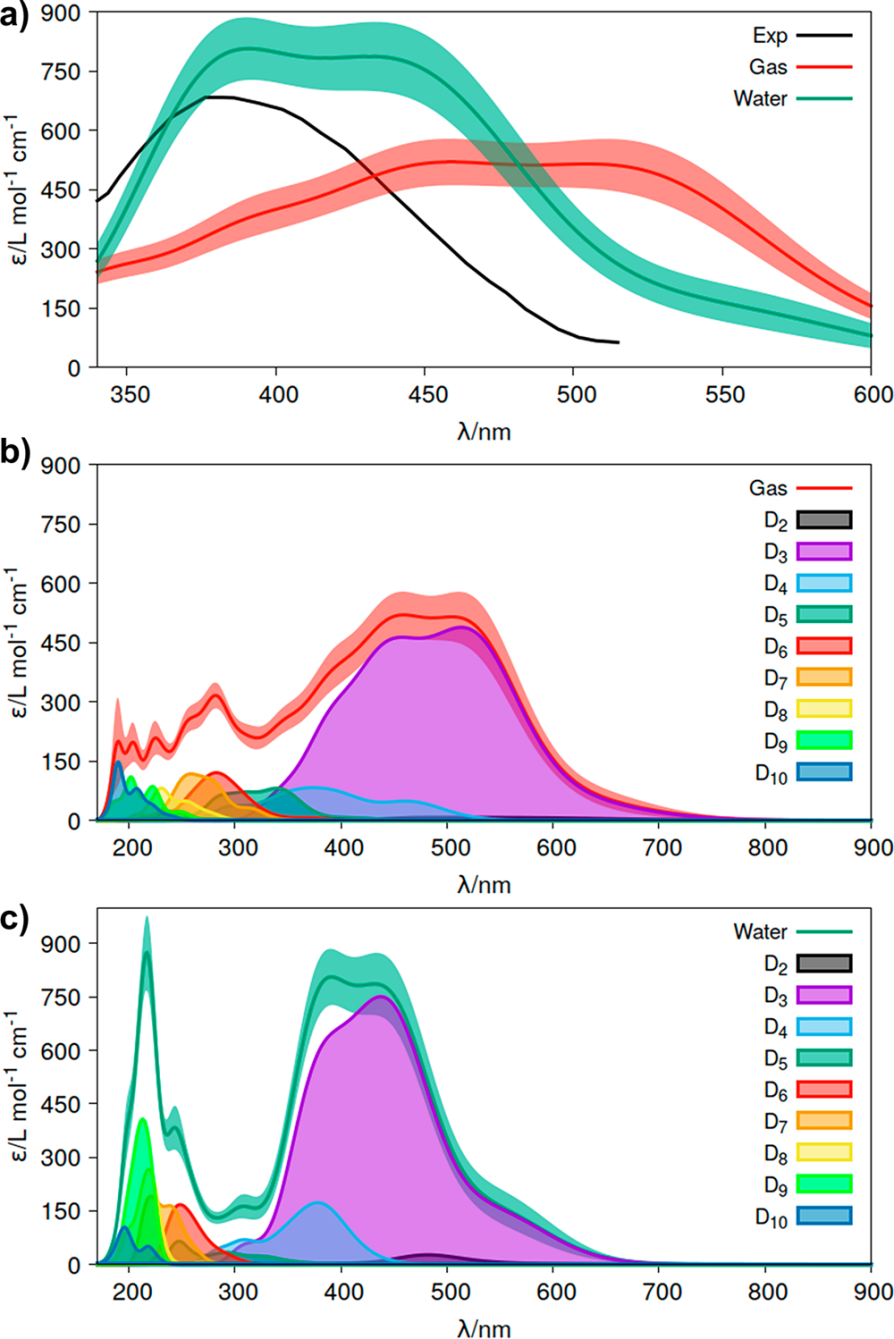
(a) Absorption molar extinction coefficients (ε) of the radical formed upon attachment of an OH radical at the C6 position of the uracil molecule (U6OH^•^) computed in the gas phase and in a water solution and absorption spectra of the transient species produced^67^ experimentally in an aqueous medium after water radiolysis in the presence of the uracil nucleobase. Shadow areas represent the statistical error of the approach (δε(*E*)). (b) Deconvolution of the absorption spectrum in the gas phase related to the electronic transitions between the ground state of the system (D_1_) and the excited states (D_2_–D_10_). (c) Analogous deconvolution of the spectrum in water.

Figure 6a reports ε(λ) in the gas phase and water solution, together with the experimental spectrum. A shift toward higher energies is found from gas to water conditions. This shift was not evident from the analyses of the dipole moments and arises from rotations of the C–OH bond with respect to the plane of the six-membered ring and from the distinct interactions of these conformations with the solvation shell.^68^ In both the gas phase and water solution, deconvolution of the transient band for all the computed doublet excited states (D_2_ to D_10_) shows that the band in the visible wavelength range is generated primarily by the D_3_ state, with a minor contribution of the D_4_ doublet at the high-energy part of the band (see Figure 6b and c). Overall, the agreement between the experimental and computed spectra in a water solution is fairly good, once again highlighting the importance of a complete sampling when computing the optical properties of chromophores.

## Summary and Conclusions

Multiconfigurational quantum chemistry (complete-active-space self-consistent field second-order perturbation theory, CASPT2) was used in this work together with distinct strategies of nuclear sampling of the chromophore and conformational and statistical sampling of the environment to determine σ(λ) in the gas phase and ε(λ) in condensed phases (water solution, interfaces or surfaces, protein or nucleic acids embeddings, etc.), focusing on the general band line shapes and avoiding rovibrational resolution. For the gas phase, molecular dynamics (MD), with a parametrized force field for the chromophore, ab initio molecular dynamics (AIMD), using density functional theory (DFT) for the electronic part of the chromophore, and Wigner sampling (WS), obtained with DFT from the optimized ground-state equilibrium structure and computed frequencies, were used. For the condensed phases, analogously, molecular dynamics (MD), quantum mechanics/molecular mechanics molecular dynamics (QM/MM MD), with DFT for the chromophore and MM for the solvent, and WS+MD, with Wigner sampling for the chromophore and MD for the solvent simulations were performed.

The computational strategies were first applied to determine the absorption spectra of acrolein in the gas phase and water solution. The blue shift of the *n*π* electronic band of this molecule experimentally measured from gas phase to water solution was used to evaluate the performance of MD, QM/MM MD, and WS+MD approaches. WS and WS+MD produce broader bands with intermediate intensities closer to the experimental data. Determination of ε(λ) at the gas phase–water interface shows an electronic band with all the features of that in the gas phase plus a shoulder at high energies attributed to the interactions of the chromophore with water molecules.

WS was also applied to determine σ(λ) and ε(λ) of the uracil radical produced by attaching OH at the C6 position of the molecule (U6OH^•^). The absorption band simulated herein agrees with the experimental spectrum of the transient species produced upon water radiolysis of uracil aqueous solution by Hayon and Simic.^67^ This supports previous assignments of the experimental band to the U6OH^•^ radical rather than the one formed by the attachment at the C5 position (U5OH^•^).^68^

Overall, the present work offers an extension of commonly used computational protocols to compute absorption electronic spectra in condensed phases based on single-reference quantum chemistry to strongly electron correlated problems considering the conformational and statistical sampling of the solvent and the nuclear quantum effects of the chromophore and with a particular focus on determining absolute values of molar extinction coefficients (ε(λ)). The approach includes the development of a mathematical program that allows the best fitting of a solute into an environment cavity, useful for an efficient and effective application. We have also extended the study to a chromophore partially solvated on a gas-phase/water interface, a situation of great importance for example in atmospheric chemistry among other fields.

## Supporting Information Available

Mathematical basis of the program *lstrans*; distribution functions of relevant internal coordinates for the nuclear samplings of acrolein in the gas phase and in water solution; radial distribution function between acrolein and water; normalized absorption intensities of acrolein reconvoluted with δ = 0.001 for the gas phase and in water solution (PDF)
