# Supplementary material for: Multiconfigurational Quantum Chemistry Determinations of Absorption Cross Sections (σ) in the Gas Phase and Molar Extinction Coefficients (ε) in Aqueous Solution and Air–Water Interface
Source: J Chem Theory Comput. 2021 May 11;17(6):3571–82. doi: 10.1021/acs.jctc.0c01083 (PMC8444339; doi:10.1021/acs.jctc.0c01083)
Supplement: Supplementary file 1 — ct0c01083_si_001.pdf [file ct0c01083_si_001.pdf]

Supporting Information

for

Multiconfigurational quantum chemistry determinations of absorption cross sections (σ) in the gas phase and molar extinction coefficients (ε) in aqueous solution and air-water interface

*Ana Borrego-Sánchez,[†,#] Madjid Zemmouche,[‡,#] Javier Carmona-García,[&] Antonio Francés-Monerris,[§,@] Pep Mulet,[‖] Isabelle Navizet,[‡] and Daniel Roca-Sanjuán[&,]**

[†] Instituto Andaluz de Ciencias de la Tierra, CSIC-University of Granada, Av. de las Palmeras 4, 18100 Armilla, Granada, Spain

[‡] MSME, Univ Gustave Eiffel, CNRS UMR 8208, Univ Paris-Est Créteil, F-77454 Marne-la-Vallée, France

[&] Instituto de Ciencia Molecular, Universitat de València, P.O.Box 22085 València, Spain

[§] Université de Lorraine and CNRS, LPCT UMR 7019, F-54000 Nancy, France
[@] Departamento de Química Física, Universitat de València, C/ Dr. Moliner 50, 46100 Burjassot, Spain

[‖] Departamento de Matemáticas Área de Matemática Aplicada Facultad de Matemáticas C/ Dr. Moliner, 50 46100 Burjassot, Spain

* Corresponding author (Daniel.Roca@uv.es)

[#] These authors contributed equally.

Table of Contents

Mathematical basis of the lstrans program……………………………………………………3

Supplementary analyses for the gas phase in acrolein

Bond lengths normalized distribution functions………………………………………...6

Absorption intensities re-convoluted with δ = 0.001…………………………………….. 9

Dihedral angles normalized distribution functions………………………………………10

Supplementary analyses for the condensed phase in acrolein

Radial distribution functions……………………………………………………………..11

Bond lengths normalized distribution functions…………………………………………12

Absorption intensities re-convoluted with δ = 0.001……………………………………..15

Dihedral angles normalized distribution functions………………………………………16

Mathematical basis of the *lstrans* program

Notation. A singular value decomposition of a matrix R is $R = U\Sigma V^T$ for orthogonal matrices U, V and diagonal matrix Σ, $\Sigma_{ii} \geq 0$. The $N \times N$ identity matrix is denoted by I_N. The Euclidian norm of a vector x is denoted by $\|x\| = \sqrt{x^T x}$. Kronecker's delta is denoted by δ_{mn}.

Lemma 1 (polar decomposition). *If $R = U\Sigma V^T$ is a singular value decomposition of an invertible matrix, then $A = UV^T$ and $S = V\Sigma V^T$ are the unique orthogonal and symmetric and positive definite matrices, respectively, satisfying $R = AS$.*

Theorem 1. *Given points $p^i, q^i \in \mathbb{R}^3$, $i = 1, \ldots, N$, we define the 3×3 matrix $R = PJQ^T$, where*

$$P = \begin{bmatrix} p^1 & \ldots & p^N \end{bmatrix}, \quad Q = \begin{bmatrix} q^1 & \ldots & q^N \end{bmatrix}, \quad J = I_N - \frac{1}{N} ee^T, \quad e^T = \begin{bmatrix} 1 & \ldots & 1 \end{bmatrix} \in \mathbb{R}^N.$$

If R is invertible then the unique solution of the absolute orientation problem

$$\min_{A,b} \sum_{i=1}^{N} \|Aq^i + b - p^i\|^2 \text{ subject to } A^T A = I_3 \tag{1}$$

is given by the orthogonal matrix A provided by the polar decomposition of R and

$$b = \frac{1}{N} \sum_{i=1}^{N} (p^i - Aq^i). \tag{2}$$

Proof. For an orthogonal matrix A we take into account that

$$\|Aq^i\|^2 = (Aq^i)^T Aq^i = (q^i)^T A^T Aq^i = (q^i)^T q^i = \|q^i\|^2$$

to simplify the objective function of the problem:

$$\sum_{i=1}^{N} \|Aq^i + b - p^i\|^2 = \sum_{i=1}^{N} \Big(\|Aq^i\|^2 + \|p^i\|^2 + \|b\|^2 - 2(p^i)^T Aq^i + 2b^T(Aq^i - p^i) \Big)$$

$$= \sum_{i=1}^{N} \Big(\|q^i\|^2 + \|p^i\|^2 - 2(p^i)^T Aq^i + \|b\|^2 + 2b^T(Aq^i - p^i) \Big) = K + \Phi(A, b),$$

where we have used the notation $K = \sum_{i=1}^{N} (\|p^i\|^2 + \|q^i\|^2)$ and

$$\Phi(A, b) = -2 \sum_{i=1}^{N} (p^i)^T Aq^i + N\|b\|^2 + 2 \sum_{i=1}^{N} b^T(Aq^i - p^i)$$

$$\Phi(A, b) = -2 \sum_{i=1}^{N} \sum_{j=1}^{3} \sum_{k=1}^{3} p_{ji} a_{jk} q_{ki} + N \sum_{j=1}^{3} b_j^2 + 2 \sum_{i=1}^{N} \sum_{j=1}^{3} b_j (\sum_{k=1}^{3} a_{jk} q_{ki} - p_{ji}). \tag{3}$$

Therefore, the minimization problem (1) is equivalent to

$$\min_{A,b} \Phi(A, b) \tag{4}$$

$$\text{subject to } \sum_{k=1}^{3} a_{ki} a_{kj} = \delta_{ij}, 1 \leq i \leq j \leq 3. \tag{5}$$

Since, for fixed A, $\lim_{\|b\| \to \infty} \Phi(A, b) = \infty$ (due to the presence of the quadratic term $N \sum_{j=1}^{3} b_j^2$), $\Phi(A, b) \geq 0$ and the set of orthogonal matrices is compact (closed and bounded) it can be seen that problem (1) has solutions. Since the 6 restrictions (5), have a rank 6 Jacobian matrix, any local minimizer of problem (1) will be a critical point of the Lagrangian of the problem, so the problem reduces to finding the minimum of the function among such critical points.

The Lagrangian of (4)-(5) is

$$\mathcal{L}(A, b, \lambda) = \Phi(A, b) + \sum_{i=1}^{3} \sum_{j=i}^{3} \lambda_{i,j} (\sum_{k=1}^{3} a_{ki} a_{kj} - \delta_{ij}),$$

with λ_{ij} denoting the Lagrange multiplier corresponding to the restrictions. From (3) the equations for the critical points of the Lagrangian corresponding to b_m, $m = 1, 2, 3$, are

$$0 = \frac{\partial \mathcal{L}}{\partial b_m} = \frac{\partial \Phi}{\partial b_m} = 2Nb_m + 2\sum_{i=1}^{N}(\sum_{k=1}^{3} a_{mk}q_{ki} - p_{mi}) \Rightarrow b_m = \frac{1}{N}\left(\sum_{i=1}^{N}\Big(p_{mi} - \sum_{k=1}^{3} a_{mk}q_{ki}\Big)\right),$$

that is, for a critical point of the Lagrangian (A, b), the vector b is determined by the orthogonal matrix A as

(6) $$b = \frac{1}{N}(P - AQ)e = \frac{1}{N}\sum_{i=1}^{N}(p^i - Aq^i),$$

which is precisely (2).

Likewise, we compute $\frac{\partial \mathcal{L}}{\partial a_{mn}}$, for $m, n = 1, 2, 3$:

$$\frac{\partial \Phi}{\partial a_{mn}} = -2\sum_{i=1}^{N} p_{mi}q_{ni} + 2\sum_{i=1}^{N} b_m q_{ni} = (-2PQ^T + 2be^TQ^T)_{mn}$$

$$\frac{\partial}{\partial a_{mn}}\Big(\sum_{i=1}^{3}\sum_{j=i}^{3}\lambda_{i,j}(\sum_{k=1}^{3} a_{ki}a_{kj} - \delta_{ij})\Big) = \sum_{i=1}^{3}\sum_{j=i}^{3}\lambda_{i,j}\sum_{k=1}^{3}(\frac{\partial a_{ki}}{\partial a_{mn}}a_{kj} + a_{ki}\frac{\partial a_{kj}}{\partial a_{mn}})$$

$$= \sum_{i=1}^{3}\sum_{j=i}^{3}\lambda_{i,j}\sum_{k=1}^{3}(\delta_{km}\delta_{in}a_{kj} + a_{ki}\delta_{km}\delta_{jn}) = \sum_{i=1}^{3}\sum_{j=i}^{3}\lambda_{i,j}(\delta_{in}a_{mj} + a_{mi}\delta_{jn})$$

$$= \sum_{j=n}^{3}\lambda_{nj}a_{mj} + \sum_{i=1}^{n}\lambda_{in}a_{mi} = 2\sum_{k=1}^{3} a_{mk}d_{kn} = 2(AD)_{mn},$$

where the symmetric matrix D is given by

(7) $$d_{kn} = \begin{cases} \frac{\lambda_{kn}}{2} & k < n \\ \lambda_{nn} & k = n \\ \frac{\lambda_{nk}}{2} & k > n. \end{cases}$$

Therefore

$$0 = \frac{\partial \mathcal{L}}{\partial a_{mn}} = (-2PQ^T + 2be^TQ^T + 2AD)_{mn}.$$

Since this is valid for any $m, n = 1, 2, 3$, it is equivalent to

$$0 = -2PQ^T + 2be^TQ^T + 2AD \Leftrightarrow be^TQ^T + AD = PQ^T.$$

If we substitute (6) we get

$$PQ^T = be^TQ^T + AD = \frac{1}{N}(P - AQ)ee^TQ^T + AD \Leftrightarrow$$

$$PQ^T - \frac{1}{N}Pee^TQ^T = A(D - \frac{1}{N}Qee^TQ^T) \Leftrightarrow$$

(8) $$R = PJQ^T = AS, \quad S = (D - \frac{1}{N}Qee^TQ^T),$$

with S being symmetric since D is so.

We have therefore established that if (A, b) is a critical points of the Lagrangian then there exist a symmetric matrix S such that

(9) $$AS = R,$$

and b given from A by (2). Since the argument is reversible, for the Lagrange multipliers can be recovered from equation (8) and (7), the critical points of the Lagrangian are characterized by (9) and (2).

The polar decomposition in Lemma 1 provides an instance of such a decomposition. The purpose of the remainder of the proof is to show that it attains the least value of the objective function among all critical points. The first step is to rewrite the objective function of (1) for an orthogonal matrix A and b given from (6).

Since for $i = 1, \dots, N$

$$Aq^i + b - p^i = Aq^i + \frac{1}{N}\sum_{j=1}^{N}(p^j - Aq^j) - p^i = A\widetilde{q}^i - \widetilde{p}^i,$$

$$\widetilde{p}^i = p^i - \frac{1}{N}\sum_{j=1}^{N} p_j = p^i - \frac{1}{N}Pe \quad \widetilde{q}^i = q^i - \frac{1}{N}\sum_{j=1}^{N} q_j = q^i - \frac{1}{N}Qe$$

we have

$$\sum_{i=1}^{N}\|Aq^i + b - p^i\|^2 = \sum_{i=1}^{N}\|A\widetilde{q}^i - \widetilde{p}^i\|^2 = \sum_{i=1}^{N}\|A\widetilde{q}^i\|^2 + \|\widetilde{p}^i\|^2 - 2(\widetilde{p}^i)^T A\widetilde{q}^i$$

$$= K_1 - 2\sum_{i=1}^{N}(\widetilde{P}^T A\widetilde{Q})_{ii} = K_1 - 2\,\mathrm{trace}(\widetilde{P}^T A\widetilde{Q}), \quad K_1 = \sum_{i=1}^{N}\|\widetilde{q}^i\|^2 + \|\widetilde{p}^i\|^2.$$

where

$$\widetilde{P} = \begin{bmatrix}\widetilde{p}^1 & \dots & \widetilde{p}^N\end{bmatrix} = P - \frac{1}{N}Pee^T = PJ, \quad \widetilde{Q} = \begin{bmatrix}\widetilde{q}^1 & \dots & \widetilde{q}^N\end{bmatrix} = Q - \frac{1}{N}Qee^T = QJ.$$

From standard properties of the trace operator we deduce

$$\mathrm{trace}(\widetilde{P}^T A\widetilde{Q}) = \mathrm{trace}((\widetilde{P}^T A\widetilde{Q})^T) = \mathrm{trace}(\widetilde{Q}^T A^T\widetilde{P}) = \mathrm{trace}(\widetilde{P}\widetilde{Q}^T A^T) = \mathrm{trace}(RA^T),$$

since

$$\widetilde{P}\widetilde{Q}^T = PJ(QJ)^T = PJJ^TQ^T$$

$$JJ^T = J^2 = (I_N - \frac{1}{N}ee^T)^2 = I_N + (\frac{1}{N}ee^T)^2 - 2\frac{1}{N}ee^T = I_N + \frac{1}{N^2}e(e^Te)e^T - \frac{2}{N}ee^T$$

$$= I_N + \frac{1}{N^2}eNe^T - \frac{2}{N}ee^T = I_N - \frac{1}{N}ee^T = J.$$

We consider an orthogonal matrix A and a symmetric matrix S that satisfy $R = AS$ and continue with the previous computation, taking into account that the trace of two similar matrices coincides and it is given by the sum of the eigenvalues:

$$\mathrm{trace}(\widetilde{P}^T A\widetilde{Q}) = \mathrm{trace}(RA^T) = \mathrm{trace}(ASA^{-1}) = \mathrm{trace}(S) = \sum_{i=1}^{3}\lambda_i(S),$$

where $\lambda_i(S)$ are the (real) eigenvalues of the symmetric matrix S, ordered as follows

$$|\lambda_1(S)| \le |\lambda_2(S)| \le |\lambda_3(S)|.$$

From $S^2 = S^TS = S^TA^TAS = R^TR$, we get $\lambda_i(S)^2 = \lambda_i(S^2) = \lambda_i(R^TR)$, thus

$$\lambda_i(S) = \mathrm{sign}(\lambda_i(S))\sqrt{\lambda_i(R^TR)}$$

with $\sqrt{\lambda_i(R^TR)} > 0$ since R is invertible. Therefore, the objective function computed for A satisfying (9) is

$$K_1 - 2\sum_{i=1}^{3}\mathrm{sign}(\lambda_i(S))\sqrt{\lambda_i(R^TR)}$$

and it attains its minimum exactly when all eigenvalues of S are positive, i.e., when S is positive definite. □

Bond lengths normalized distribution functions for the gas phase in acrolein

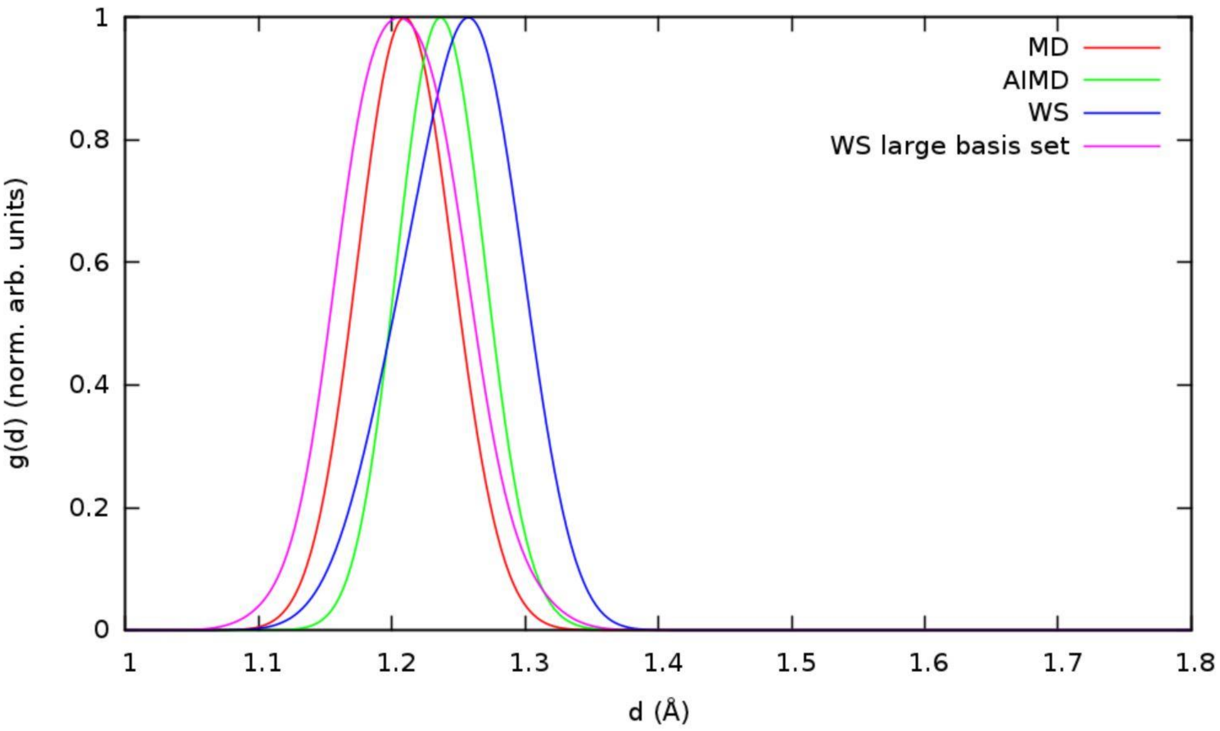

Figure S1. Normalized distribution functions (g(d)) of the O=C double bond length (d) from the ensemble of geometries obtained for acrolein in the gas phase with distinct sampling schemes: classical molecular dynamics (MD), ab initio molecular dynamics (AIMD), Wigner sampling with the 6-31G basis set (WS) and Wigner sampling with the larger basis set 6-311G(2d,p) (WS large basis set).

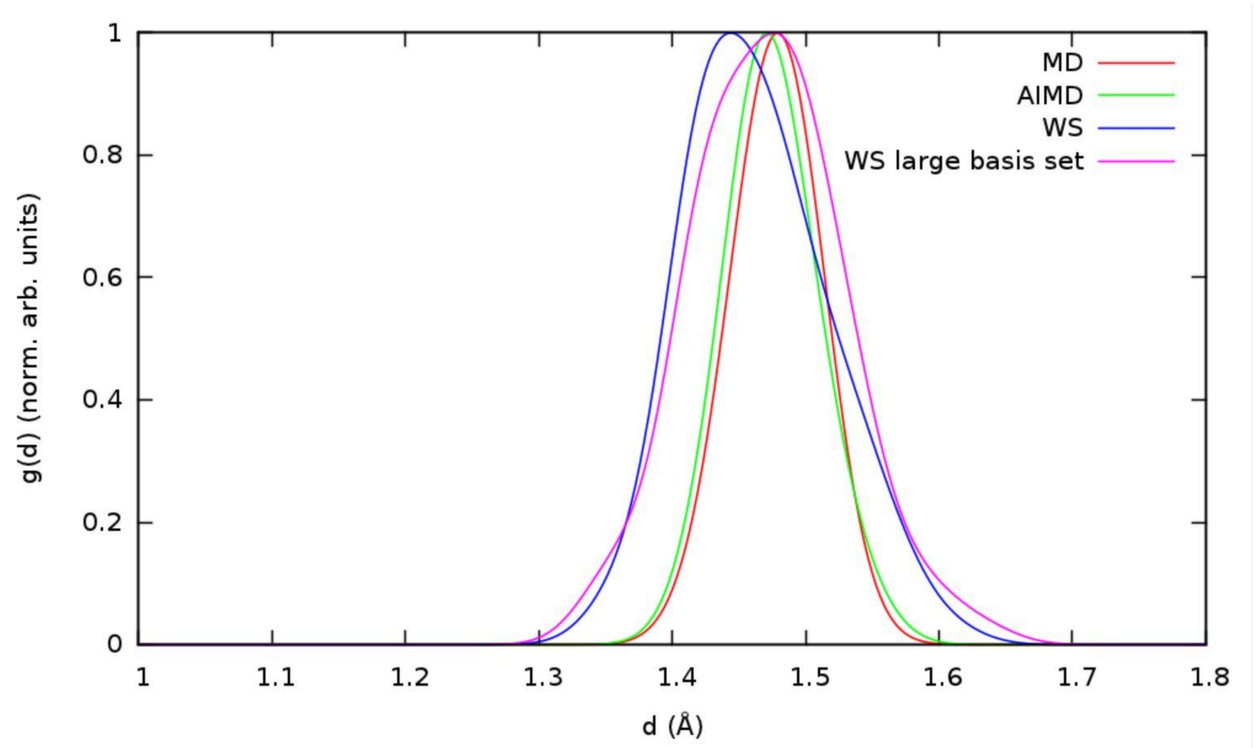

Figure S2. Normalized distribution functions (g(d)) of the C-C single bond length (d) from the ensemble of geometries obtained for acrolein in the gas phase with distinct sampling schemes: classical molecular dynamics (MD), ab initio molecular dynamics (AIMD), Wigner sampling with the 6-31G basis set (WS) and Wigner sampling with the larger basis set 6-311G(2d,p) (WS large basis set).

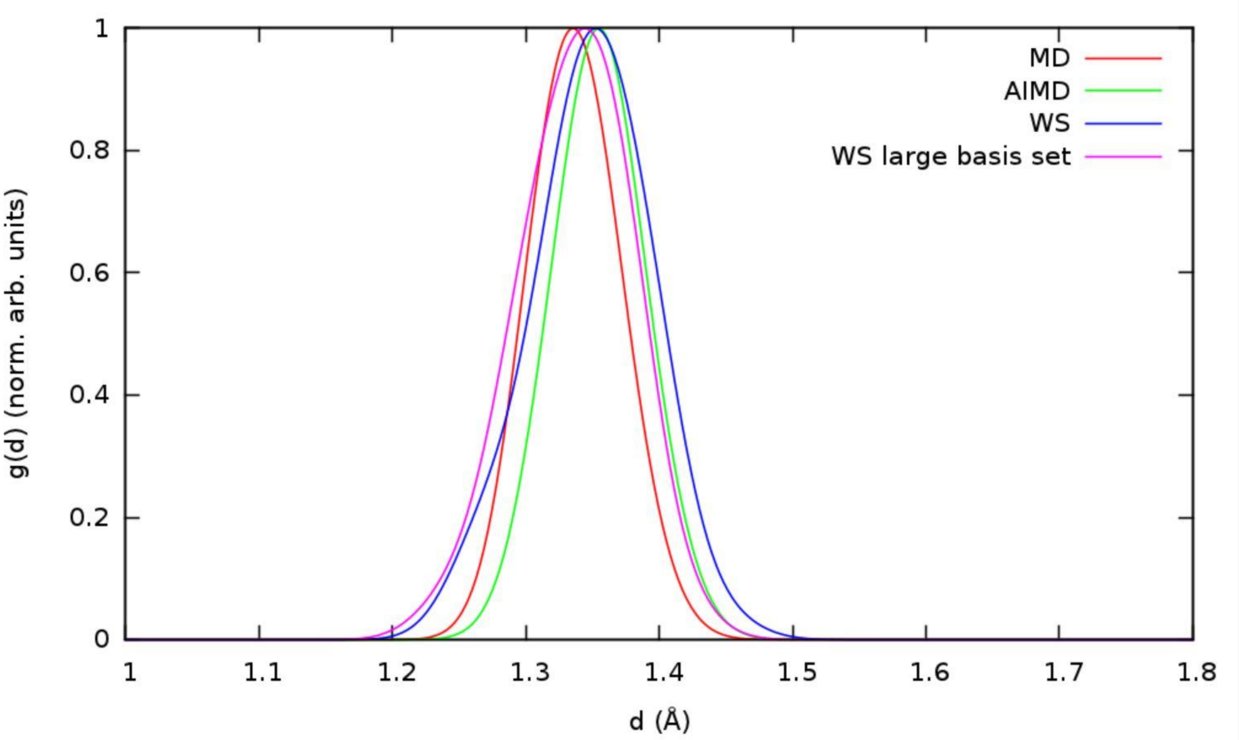

Figure S3. Normalized distribution functions (g(d)) of the C=C double bond length (d) from the ensemble of geometries obtained for acrolein in the gas phase with distinct sampling schemes: classical molecular dynamics (MD), ab initio molecular dynamics (AIMD), Wigner sampling with the 6-31G basis set (WS) and Wigner sampling with the larger basis set 6-311G(2d,p) (WS large basis set).

Absorption intensities of acrolein in the gas phase re-convoluted with δ = 0.001

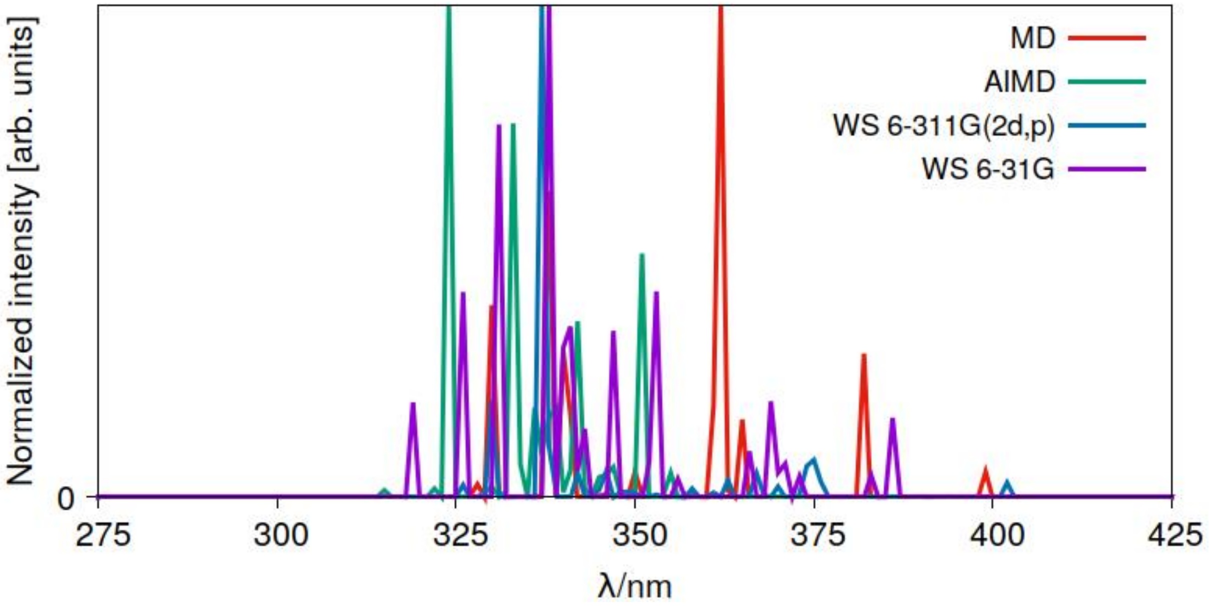

Figure S4. Normalized absorption intensities of acrolein in the gas phase computed at the CASPT2(6,5)/ANO-L-VTZP level of theory using distinct conformational sampling approaches, classical molecular dynamics (MD), ab initio molecular dynamics (AIMD), and Wigner sampling (WS). For WS, two basis sets are compared, 6-31G (WS 6-31G) and 6-311G(2d,p) (WS 6-311G(2d,p)) and using a reduced phenomenological broadening of δ = 0.001. The normalization is performed for each set of sampling data.

Dihedral angles normalized distribution functions for the gas phase in acrolein

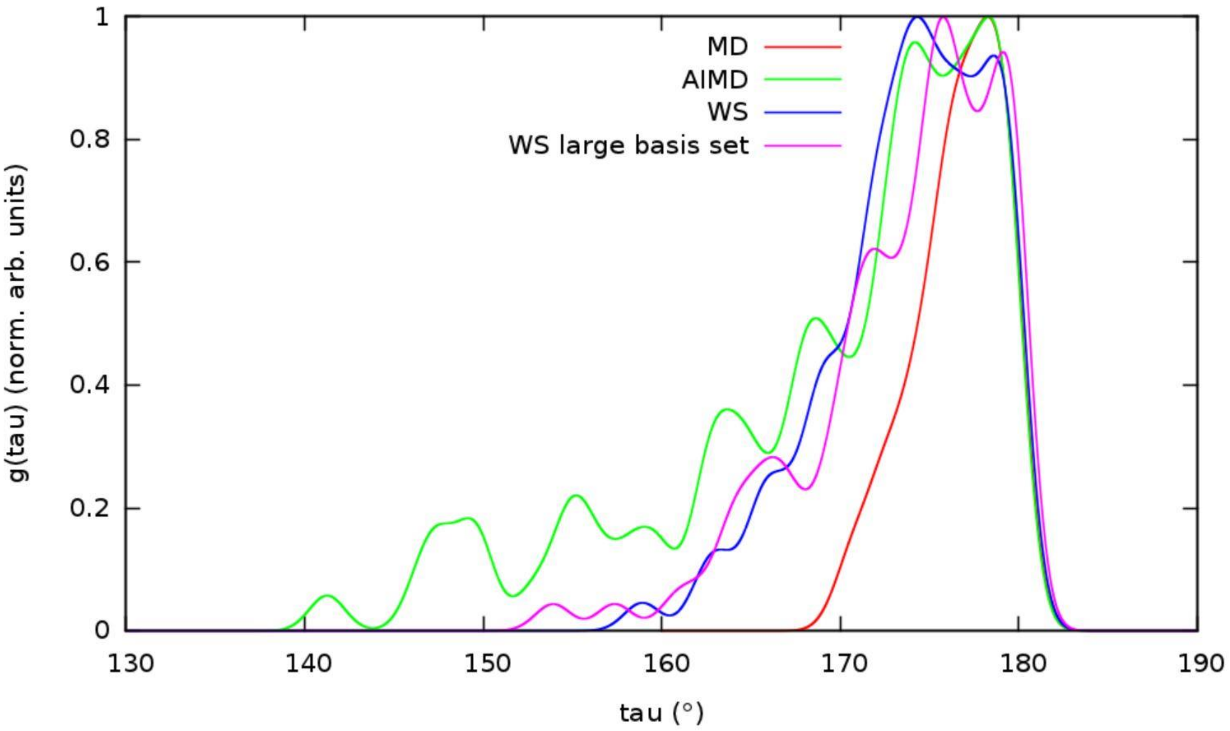

Figure S5. Normalized distribution functions (g(τ)) of the O=C-C=C dihedral angle (τ), in absolute value, from the ensemble of geometries obtained for acrolein in the gas phase with distinct sampling schemes: classical molecular dynamics (MD), ab initio molecular dynamics (AIMD), Wigner sampling with the 6-31G basis set (WS) and Wigner sampling with the larger basis set 6-311G(2d,p) (WS large basis set).

Radial distribution functions for water solution in acrolein

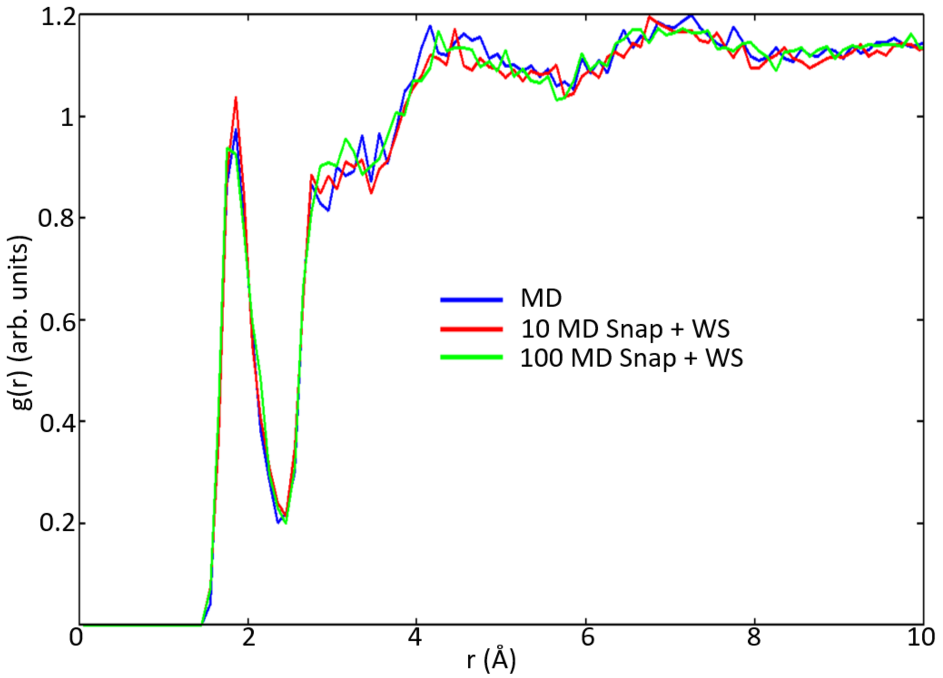

Figure S6. Radial distribution function (g(r)) between the acrolein atoms and those of the water solvent molecules for the 100 structures obtained in the 1 ns classical MD of Amber14 (blue line) and the 100 geometries generated by inserting the Wigner geometries of the chromophore into 10 or 100 snapshots (red and green lines, respectively) from the 1 ns classical MD of Amber14 and relaxing the system with 10 ps MD (see text).

Bond lengths normalized distribution functions for the condensed phase in acrolein

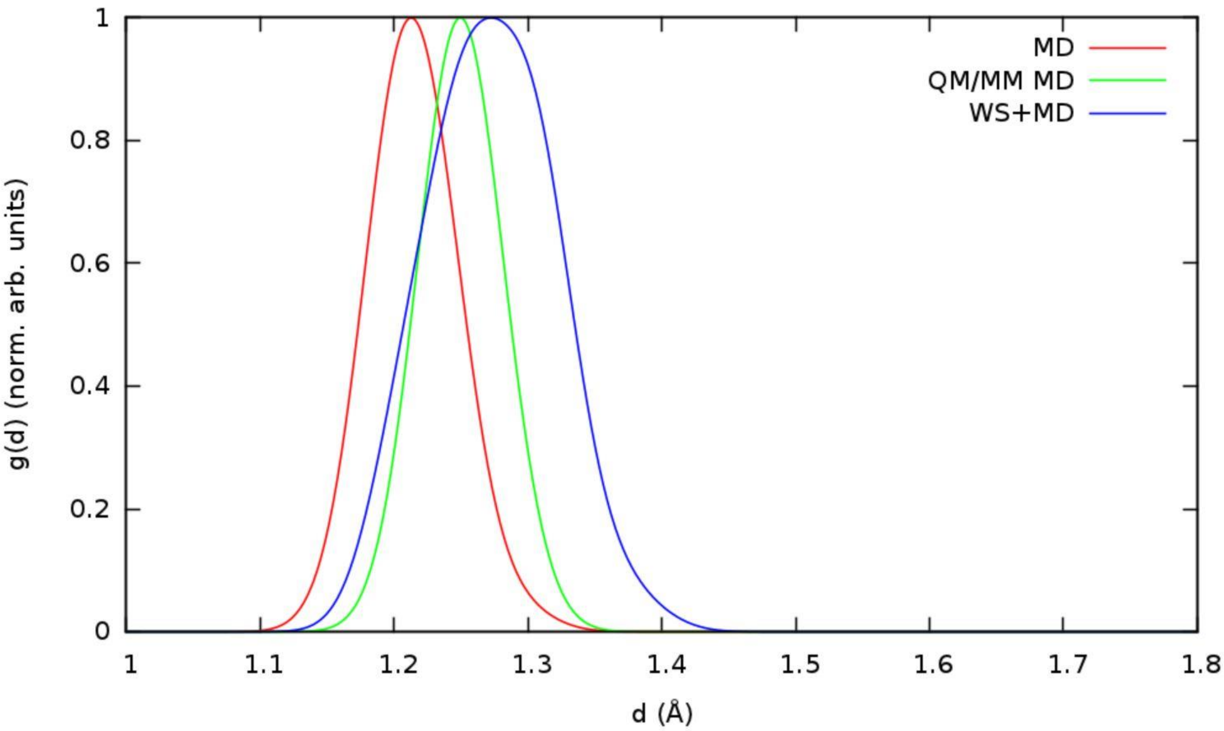

Figure S7. Normalized distribution functions (g(d)) of the O=C double bond length (d) from the ensemble of geometries obtained for acrolein in water solution with distinct sampling schemes: classical molecular dynamics (MD), quantum mechanics / molecular mechanics molecular dynamics (QM/MM MD) and mixed Wigner sampling with the 6-31G basis set (chromophore) and MD sampling for the solvent (WS+MD). Note that for the last approach the chromophore geometries are the same for the two independent MD simulations carried out with Amber14 and Materials Studio 2019.

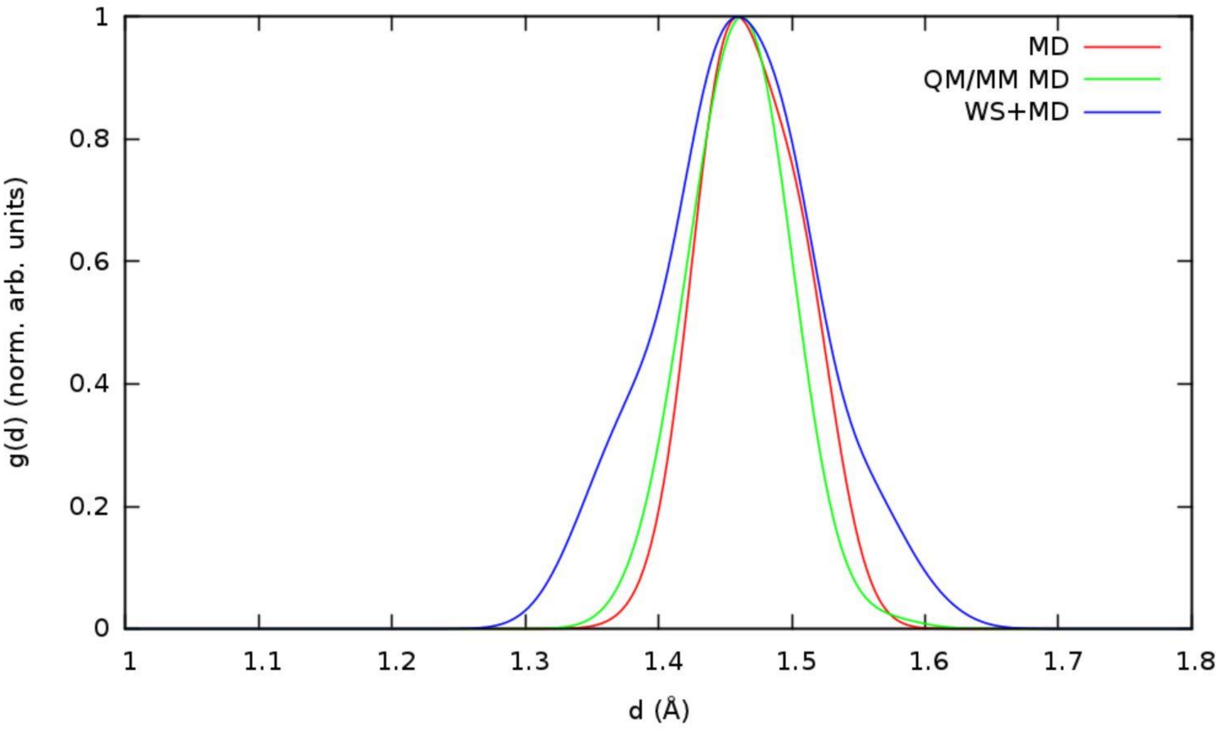

Figure S8. Normalized distribution functions (g(d)) of the C-C single bond length (d) from the ensemble of geometries obtained for acrolein in water solution with distinct sampling schemes: classical molecular dynamics (MD), quantum mechanics / molecular mechanics molecular dynamics (QM/MM MD) and mixed Wigner sampling with the 6-31G basis set (chromophore) and MD sampling for the solvent (WS+MD). Note that for the last approach the chromophore geometries are the same for the two independent MD simulations carried out with Amber14 and Materials Studio 2019.

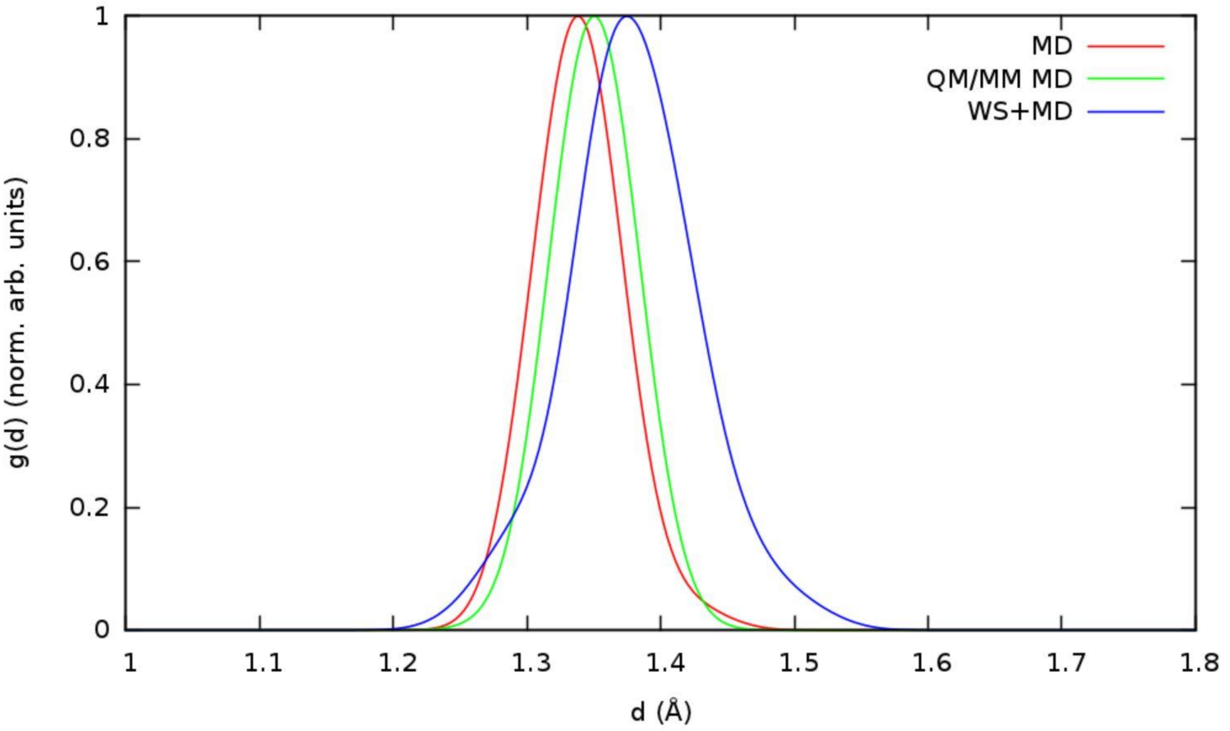

Figure S9. Normalized distribution functions (g(d)) of the C=C double bond length (d) from the ensemble of geometries obtained for acrolein in water solution with distinct sampling schemes: classical molecular dynamics (MD), quantum mechanics / molecular mechanics molecular dynamics (QM/MM MD) and mixed Wigner sampling with the 6-31G basis set (chromophore) and MD sampling for the solvent (WS+MD). Note that for the last approach the chromophore geometries are the same for the two independent MD simulations carried out with Amber14 and Materials Studio 2019.

Absorption intensities of acrolein in the condensed phase re-convoluted with δ = 0.001

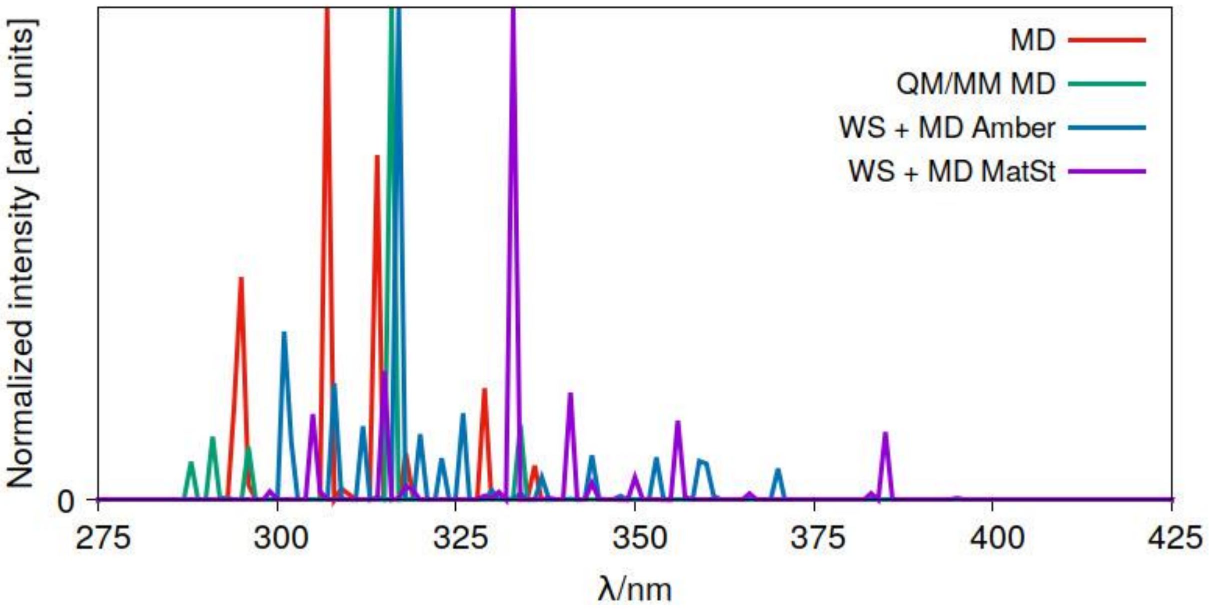

Figure S10. Normalized absorption intensities of acrolein in the gas phase computed at the CASPT2(6,5)/ANO-L-VTZP level of theory using distinct conformational sampling approaches, classical molecular dynamics (MD), quantum mechanics / molecular mechanics molecular dynamics (QM/MM MD), and Wigner and molecular dynamics sampling for the chromophore and solvent, respectively (WS+MD), and using a reduced phenomenological broadening of δ = 0.001. For WS+MD, two implementations are compared, Amber (WS+MD Amber) and Materials Studio (WS+MD MatSt). The normalization is performed for each set of sampling data.

Dihedral angles normalized distribution functions for the condensed phase in acrolein

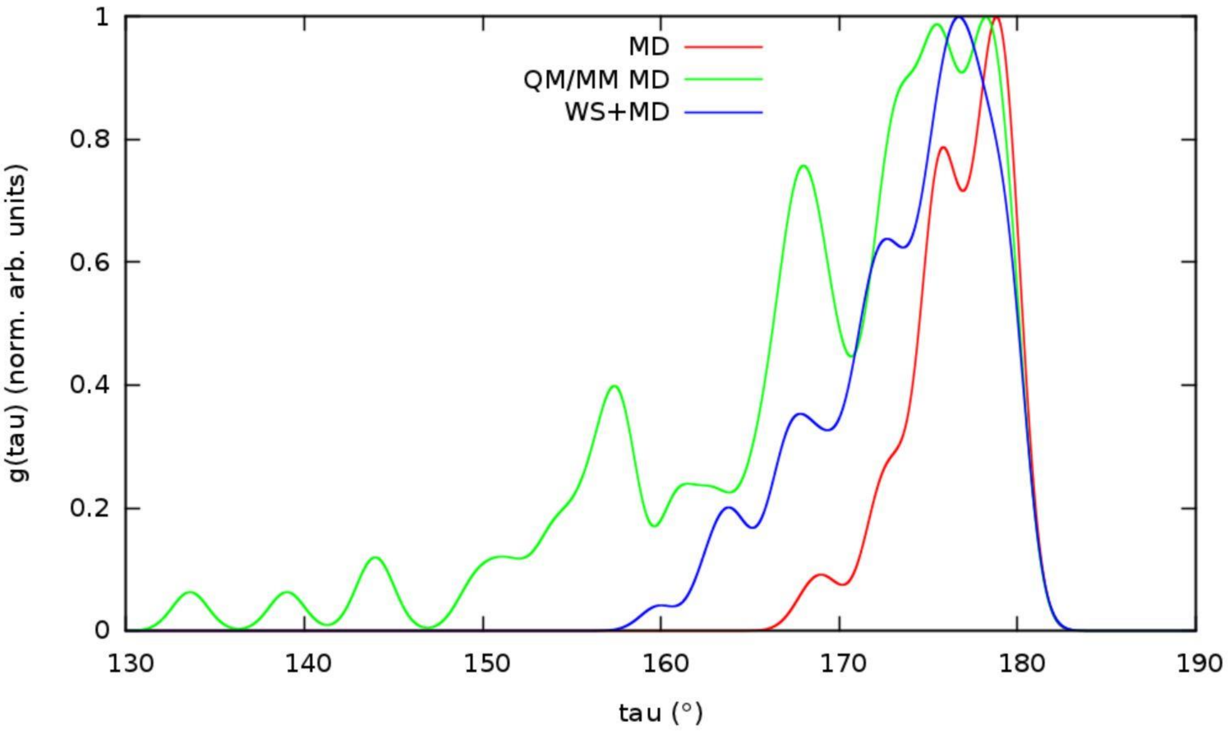

Figure S11. Normalized distribution functions (g(τ)) of the O=C-C=C dihedral angle (τ), in absolute value, from the ensemble of geometries obtained for acrolein in water solution with distinct sampling schemes: classical molecular dynamics (MD), quantum mechanics / molecular mechanics molecular dynamics (QM/MM MD) and mixed Wigner sampling with the 6-31G basis set (chromophore) and MD sampling for the solvent (WS+MD). Note that for the last approach the chromophore geometries are the same for the two independent MD simulations carried out with Amber14 and Materials Studio 2019.
